# Long-Term Effects of COVID-19 and the Pandemic on Tinnitus Patients

**DOI:** 10.3389/fneur.2022.921173

**Published:** 2022-06-30

**Authors:** Murat Erinc, Ahmet Mutlu, Serdal Celik, Mahmut Tayyar Kalcioglu, Agnieszka J. Szczepek

**Affiliations:** ^1^Department of Audiology, Faculty of Health Sciences, Istanbul Medeniyet University, Istanbul, Turkey; ^2^Department of Otorhinolaryngology, Faculty of Medicine, Istanbul Medeniyet University, Istanbul, Turkey; ^3^Goztepe Prof. Dr. Suleyman Yalcin City Hospital, Istanbul, Turkey; ^4^Department of Otorhinolaryngology, Head and Neck Surgery, Charité-Universitätsmedizin Berlin, Corporate Member of Freie Universität Berlin and Humboldt-Universität zu Berlin, Berlin, Germany; ^5^Faculty of Medicine and Health Sciences, University of Zielona Góra, Zielona Góra, Poland

**Keywords:** tinnitus, COVID-19, THI, tinnitus-induced distress, tinnitus loudness, hyperacusis

## Abstract

This study aimed to explore the effect of COVID-19 and the pandemic period on the tinnitus-related complaints of patients with chronic tinnitus. Ninety-six patients who were diagnosed with chronic tinnitus before the pandemic were enrolled in this study. Before the pandemic and in January 2022, all patients used the Visual Analog Scale (VAS) to assess tinnitus loudness, annoyance, and effect on everyday life, sleep, and concentration. Additionally, patients filled the Tinnitus Handicap Inventory (THI) and the Hyperacusis Questionnaire (HQ). In the entire cohort, tinnitus loudness, annoyance, and tinnitus-induced difficulties with concentration as well as THI and HQ scores increased significantly during the two pandemic years. Thirty-seven tinnitus patients contracted COVID-19 between March 2020 and January 2022. These patients were asked to list leading COVID-19 symptoms, changes in tinnitus complaints during and after the disease, and whether their hearing abilities were affected. Three patients in the COVID-19 group confirmed worsening their hearing abilities. There was no decrease in the tinnitus complaint during COVID-19, 24.3% of the infected patients reported exacerbation of tinnitus, and 75.7% said tinnitus remained the same. In the COVID-19-negative group, 13.5% reported tinnitus decrease during the pandemic, 57.6% said it remained the same, and 28.8% reported exacerbation of tinnitus. When split into infected and non-infected groups, a significant increase in tinnitus loudness, tinnitus effect on concentration, and THI scores were seen only in patients who contracted COVID-19, while hyperacusis worsened significantly (*p* < 0.05) only in COVID-19-negative tinnitus patients. Despite significant differences within the groups, there were no differences found between the groups. This study points to possible different effects of the infection with SARS-CoV-2 and the pandemic period on patients with chronic tinnitus. It also provides evidence for deterioration of preexisting tinnitus as a possible long-term effect of COVID-19.

## Introduction

COVID-19 is a highly contagious sickness caused by the infection with the SARS-CoV-2 virus (1). At the end of December 2019, the outbreak of COVID-19 occurred in Wuhan, China, and then spread worldwide, causing a pandemic that continues until today. Although the symptoms and severity of COVID-19 vary, the most common symptoms are fever, dry cough, and fatigue (2). Anorexia, shortness of breath, and myalgia are often reported, while nausea and diarrhea are less common. Additionally, sore throat, rhinorrhea, nasal congestion, tonsillar hyperemia, cervical lymphadenitis, hyposmia/anosmia, and dizziness/imbalance are reported occasionally by COVID-19 patients (3).

Early during the pandemic, tinnitus was described as one of the initial symptoms of COVID-19, accounting for 3.5% of the cases (4). This observation was followed by case reports (5–7) and more extensive studies suggesting that tinnitus is a frequent complaint of patients affected by COVID-19 (8, 9). Among the 119 hospitalized SARS-CoV-2-PCR-positive patients, tinnitus comprised 11% of otolaryngological complaints (10). In addition, during the pandemic, it was observed that significantly more patients sought professional help for tinnitus in tertiary healthcare institutions than before the pandemic (11). In a large UK-based survey-based study that enclosed 6,881 subjects with a sub-sample of 1,274 individuals who had tinnitus prior to the pandemic, tinnitus worsened significantly more in those who contracted COVID-19 than in the non-infected ones. However, caution was advised when establishing a relationship between auditory symptoms and COVID-19 based on self-reported data (12).

In August 2020, a group of Italian clinicians described symptoms (particularly fatigue and dyspnea) that persisted in 87.4% of COVID-19 patients after the acute phase of illness (13). A few months later, a guideline defined “Long COVID” as “signs and symptoms that continue or develop after acute COVID-19” (14). Shortly after, an editorial published in “Nature” called for patients' help defining the symptoms of long COVID (14). In the past 2 years, tinnitus was reported as a sequel of COVID-19, suggesting it could be one of the symptoms of Long COVID. In a study of Egyptian COVID-19 patients, tinnitus occurred as a post-COVID manifestation in 16.7% of 287 subjects (mean age 32.3 ± 8.5; range 20–60) who recovered from COVID-19 (15). Another observational study of a Danish cohort that selected patients with COVID-related taste and/or smell loss determined that tinnitus occurred in 16.4% of these patients 30 days after the onset of initial symptoms (16). Remarkably, in a long-term follow-up (seven months after the recovery from COVID-19) of 31 patients included, 21 subjects reported the persistence of COVID-related tinnitus. Only 7/21 had a full recovery, while 14/21 had partial or no recovery, suggesting that tinnitus can be a long-term sequel of COVID-19.

In addition to the potential effects of SARS-CoV-2 infection on the already existing tinnitus, the pandemic situation's influence on tinnitus patients should be considered. Patients with chronic tinnitus are known to react to psychological burden or emotional stress with worsening tinnitus symptoms (17–20), and the situation created by the pandemic can be viewed as a stressor (21–25).

Two research questions prompted us to perform the study described here: (“*does the infection with SARS-CoV-2 influence already existing tinnitus or hyperacusis?*”) and (“*does the situation created by the pandemic influence already existing tinnitus or hyperacusis?*”). To address the above questions, we investigated the effect of COVID-19 on a sample of patients with chronic tinnitus. We collected and compared the tinnitus and hyperacusis-related data obtained from tinnitus patients before and 2 years after the pandemic onset. Analyses were performed for the infected and non-infected tinnitus patients.

## Materials and Methods

### Study Design and Patients

The Clinical Research Ethics Committee approved the study of Istanbul Medeniyet University (2021/0665). Between July 2018 and March 2020, a cohort of 123 patients was diagnosed with chronic subjective idiopathic tinnitus, agreeing with the international guideline for tinnitus diagnosis (26). All patients underwent medical (including radiological imaging) and audiological evaluations in the pre-pandemic period and were regularly followed up. The patients had no history of neurological disease, tumor, or brain injury. All patients were diagnosed with either bilateral or unilateral chronic idiopathic tinnitus, and 75% had no comorbid disease (Table 1).

**Table 1:** Description of 96 participants.

**Gender**	**Female** *n* **= 52; Male** *n* **= 44**		
Age (years)	Minimum 20	Maximum 86	Mean 50.8 (SD 14.5)
Tinnitus duration (months)	Minimum 25	Maximum 444	Mean 76.0 (SD 66.0)
**Laterality of tinnitus**			
	Bilateral	*n* = 53 (55.2%)	
	Right	*n* =15 (15.7%)	
	Left	*n* = 28 (29.1%)	
**Comorbidities**			
	Chronic otitis media	*n* = 3 (3.1%)	
	Otosclerosis	*n* = 1 (1%)	
	Diabetes mellitus	*n* = 3 (3.1%)	
	Hypertension	*n* = 13 (13.5%)	
	Thyroid gland diseases	*n* = 4 (4.1%)	
	None	*n* = 72 (75%)	

In January 2022, all 123 patients were contacted telephonically, and 96 patients agreed to participate in the study. Of 96 patients included in this study, 52 (54.2%) were female and 44 (45.8%) male. The mean age of the patients was 50.8 ± 14.5 (min: 20; max: 83).

**Figure 1:**
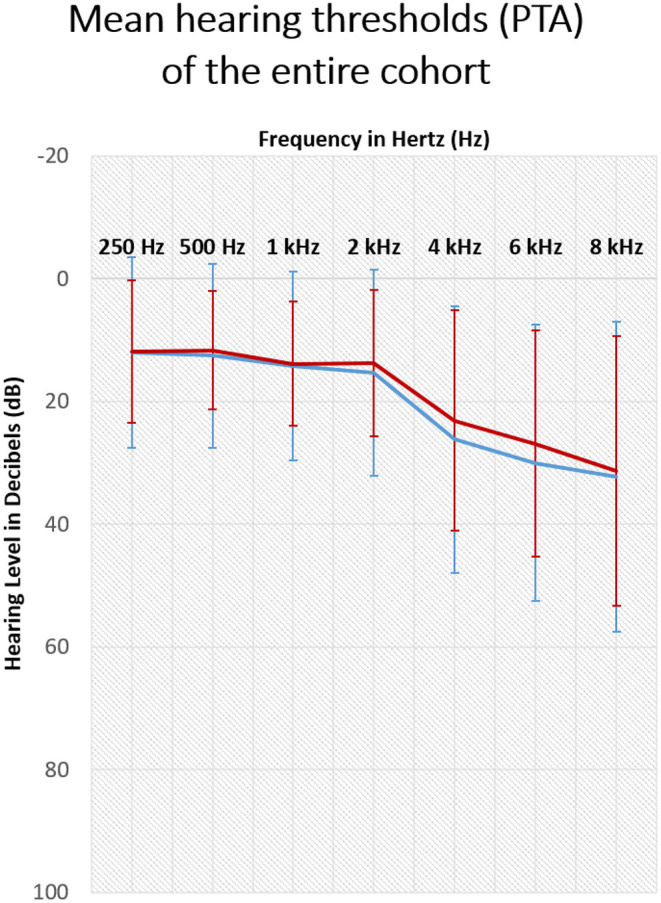
Hearing thresholds of tinnitus patients measured with pure tone audiometry (250–8,000Hz) before the pandemic. Shown are the mean results of the sample with SD. The red line: right ear, blue line: left ear.

In agreement with WHO-proposed grades of hearing impairment based on the average pure-tone hearing sensitivity at 500, 1,000, 2,000, and 4,000 Hz (27), one patient (infected) had profound hearing impairment in one ear but reported tinnitus in the contralateral ear affected only by a mild hearing loss. Another patient (uninfected) had moderately severe hearing impairment; two patients (both uninfected) had moderate hearing loss, 19 had mild hearing loss (nine infected and 10 uninfected), and 66 (23 infected and 43 uninfected) had normal hearing. No data was available for seven patients (four infected and three uninfected). The audiogram plot with mean values was sloping in higher frequencies (Figure 1). The patients diagnosed with hearing loss obtained a referral for hearing aids or CI. Three tinnitus education sessions and counseling were offered to all patients.

The telephone interview lasted, on average, 15–20 min. Thirty-seven (38.5%) of participating patients reported having contracting COVID-19.

### Questionnaires and Visual Analog Scales (VAS)

The visual analog scale (VAS) (28) assessed the tinnitus loudness, annoyance, and effect on everyday life, sleep, and concentration. The patients were instructed that 0 corresponds to a lack of symptoms (no tinnitus, no tinnitus-related annoyance, no sleep deprivation, no effect on concentration) and 10 to the worse possible symptoms (extremely loud tinnitus, extremely annoying tinnitus, tinnitus-induced insomnia, tinnitus-related inability to concentrate). In the VAS assessing the impact of tinnitus on everyday life, 0 corresponded to no effect and 10 to a highly negative effect.

Tinnitus Handicap Inventory (THI) (29) consists of 25 items with a total of 100 points, and the response choices are “no” (0 points), “sometimes” (2 points), and “yes” (4 points). The total score between 0 and 16 indicates no handicap; 18–36 mild handicap, 38–56 moderate handicap, and 58–100 points severe handicap. Turkish validated THI (30) was used in the present study.

The Hyperacusis Questionnaire (HQ) (31) consists of 14 items with a total of 42 points, and the response choices are “'no” (0 points), “yes, a little” (1 point), “yes, quite a lot” (2 points), and “yes, a lot” (3 points). The suggested cut-off value of 28 (31) was found high by many researchers, and different values were suggested to be used (32–36). In the Turkish version of the questionnaire, a score of 15 and above is recommended as “suspected hyperacusis” (37).

**Figure 2:**
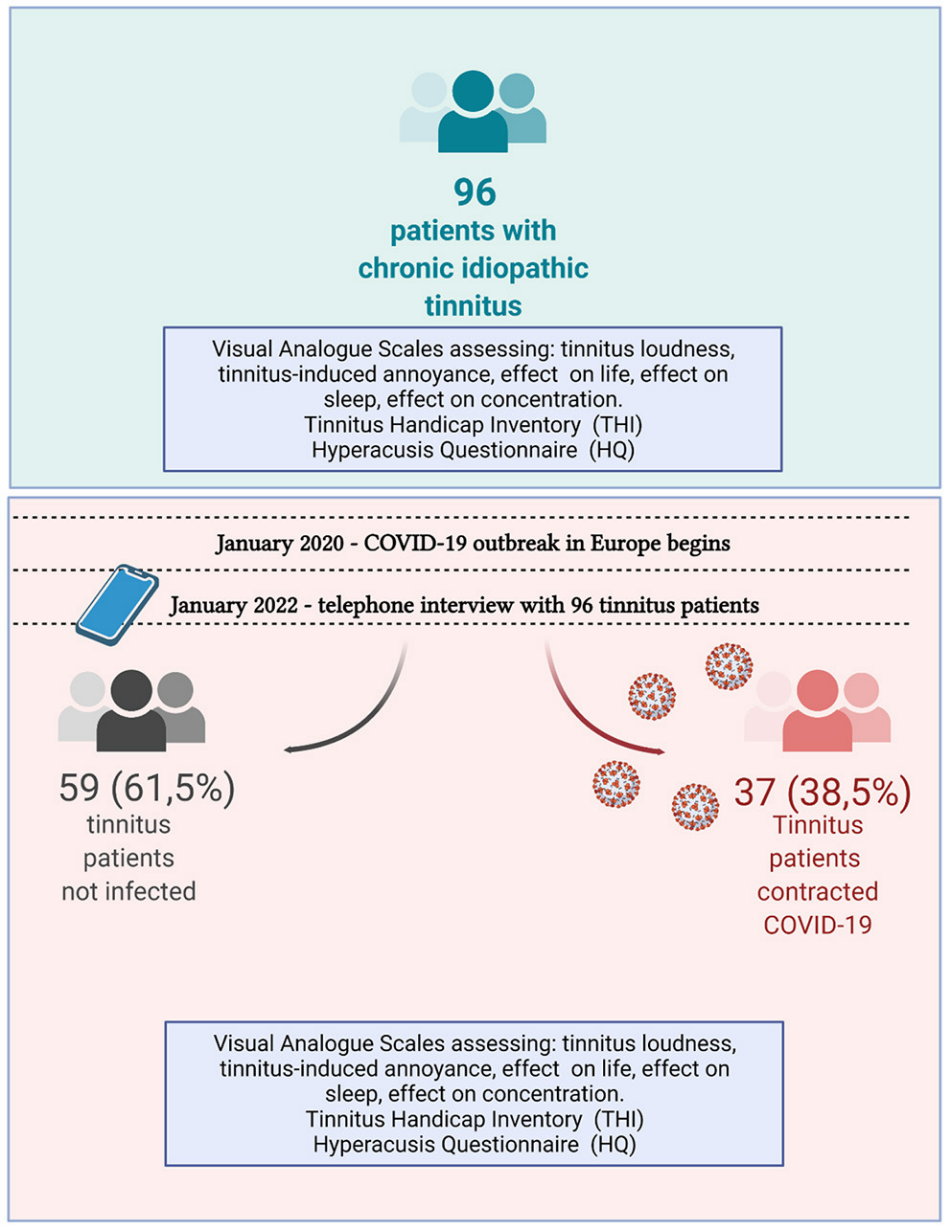
Schematic drawing showing the study design and a workflow.

All patients filled out the questionnaires prior to the pandemic. In January 2022, a single researcher conducted all surveys (Figure 2). That researcher first ensured that the patients fully understood the survey questions and then answered them. All patients were asked about changes in tinnitus and hearing complaints during the pandemic. THI, HQ, and the VAS scales were completed during the survey. In addition, patients who underwent COVID-19 were asked about COVID-19-related symptoms (open question).

### Statistical Analysis

All statistical analyzes were performed using SPSS v20 for MAC (Statistical Package for Social Science, IBM). Descriptive data of related parameters are given as mean, median, standard deviation, minimum, and maximum values. Ordinal data of patients with and without COVID-19 were compared using the Mann Whitney-U test, and the within-group comparison was performed with the Wilcoxon test. Statistical significance was set for *p* < 0.05.

## Results

Upon analysis of the entire cohort, we found significant differences in scores regarding tinnitus loudness, annoyance, problems with concentration, as well as THI and HQ scores (Wilcoxon paired test). Next, we split the cohort into two groups: 37 patients who contracted COVID-19 (38.5%) and 59 (61.5%) patients who did not. The mean duration of tinnitus complaints in patients with COVID-19 was 75 ± 63.4 months (range 27–384 months), while the mean duration of tinnitus complaints in patients without COVID-19 was 76.6 ± 68.6 months (range 25–384 months). Of 37 patients with a history of COVID-19, 15 (40.5%) were female, and 22 (59.5%) were male. Of the 59 COVID-19-negative patients, 37 (62.7%) were female, and 22 (37.3%) were male. Four patients with COVID-19 had to be admitted to a hospital (5–14 days). Non-invasive respiratory support was needed in 3 of 4 cases (10.8%) among hospitalized patients. None of the patients diagnosed with COVID-19 were hospitalized in the intensive care unit.

In response to a specific question (“*have your hearing abilities changed during COVID-19*?”), three of the four hospitalized COVID-19 patients (8.1%) stated that their hearing abilities worsened. Five patients stated that their tinnitus worsened during COVID-19 and remained worse after the illness. Three patients reported worsening tinnitus during COVID-19 but a recovery to the baseline after COVID-19. One patient reported worsening of his tinnitus during and after COVID-19. None of the patients complained about Eustachian tube dysfunction or ear fullness during or after COVID-19. The most common complaints regarding COVID-19—related leading symptoms were loss of smell (19 patients, 51.4%); cough (16 patients, 43.2%); loss of taste (14 patients, 37.8%), and shortness of breath (14 patients, 37.8%).

In the COVID-19 group, none of the patients reported a decrease in tinnitus-related distress, nine patients (24.3%) reported an increase in tinnitus complaints, and 28 patients (75.7%) reported no changes. In contrast, in the COVID-19-free group, eight (13.5%) patients stated that their tinnitus decreased, for 34 patients (57.6%), tinnitus remained the same, and 17 (28.8%) increased.

Between-group comparison of patients with and without COVID-19 has not indicated significant changes in the pre- and post-pandemic data (Table 2).

**Table 2:** Comparison between COVID-19-positive and negative tinnitus patients concerning the pre-, and post-pandemic mean scores of VAS (tinnitus loudness, annoyance, effect on life, sleep, and concentration), Tinnitus Handicap Inventory (THI), and the Hyperacusis Questionnaire (HQ).

	**Between-groups comparison of the pre- COVID-19 pandemic period comparisons**	**Between-groups comparison of the post- COVID-19 pandemic period comparisons**
VAS (tinnitus loudness)	*U* = 1,047, *p* = 0.737	*U* = 1,213, *p* = 0.355
VAS (tinnitus annoyance)	*U* = 995, *p* = 0.463	*U* = 958, *p* = 0.313
VAS (tinnitus effect on life)	*U* = 1,147, *p* = 0.671	*U* = 1,096, *p* = 0.973
VAS (tinnitus effect on sleep)	*U* = 1,026, *p* = 0.619	*U* = 1,104, *p* = 0.925
VAS (tinnitus effect on concentration)	*U* = 1,092, *p* = 0.994	*U* = 1,199, *p* = 0.413
THI	*U* = 1,030, *p* = 0.643	*U* = 1,201, *p* = 0.407
HQ	*U* = 1,209, *p* = 0.371	*U* = 950, *p* = 0.288

*The Mann–Whitney-U test results (U) and the 2-tailed significance (p) are shown. No differences between the groups were found*.

**Figure 3:**
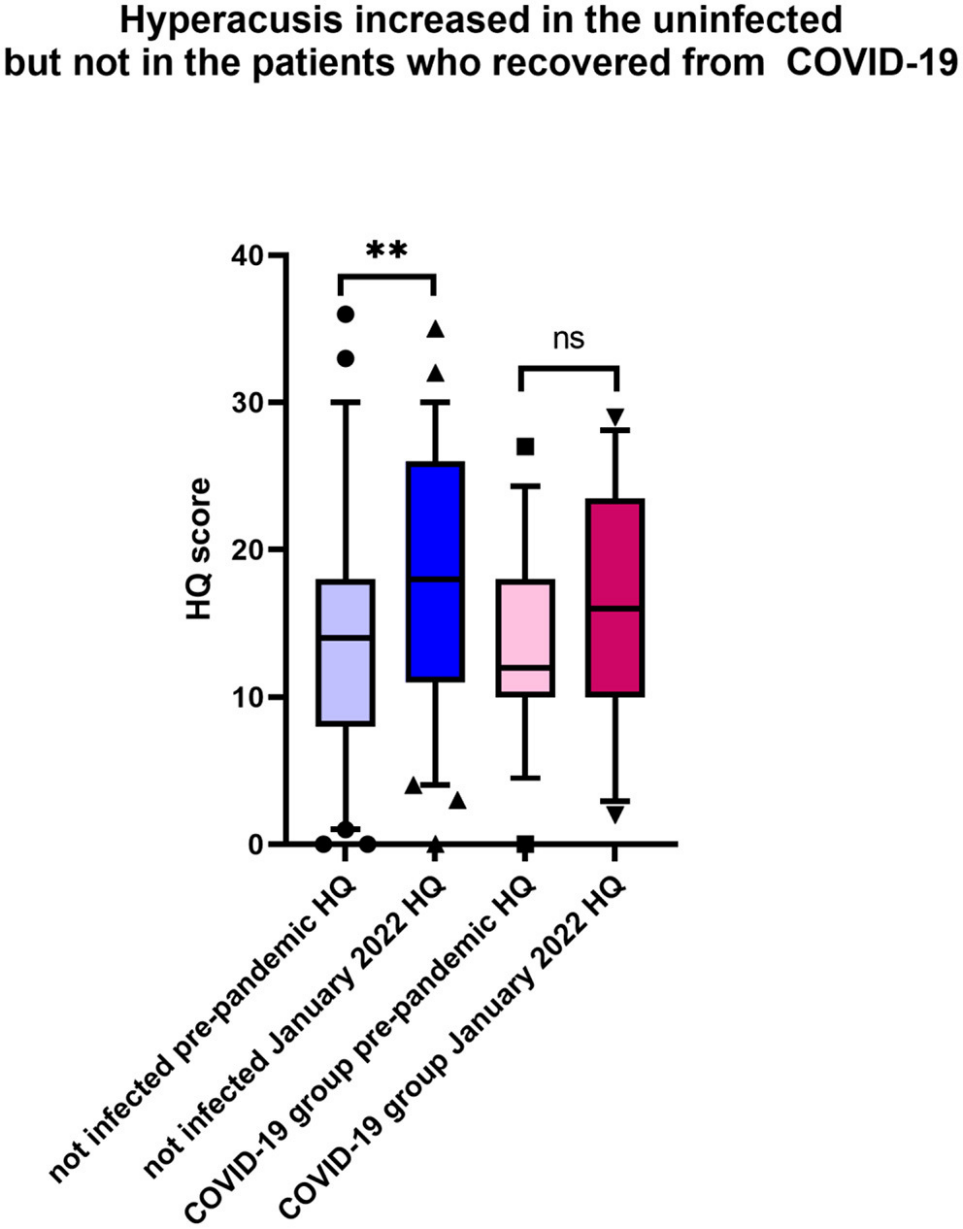
During the pandemic, hyperacusis increased in the uninfected tinnitus patients. Shown is the box & whiskers plot (5–95 percentile), Wilcoxon paired test (^**^p < 0.0001; ns, not significant).

The analysis indicated a significant worsening of hyperacusis symptoms during the pandemic in the uninfected tinnitus patients, which was not the case in the infected group (Table 3, Figure 3). Other parameters tested in the COVID-19-free group before the pandemic and in January 2022 remained unchanged.

**Table 3:** Scores of Tinnitus Handicap Inventory (THI), Hyperacusis Questionnaire (HQ), and the visual analog scales (VAS) in COVID-19-negative tinnitus patients.

***N*** **= 59**		**Pre-pandemic**	**Post-pandemic**	***p***
VAS (tinnitus loudness) (0–10)	Mean ± std dev	5.3 ± 1.9	5.7 ± 2.1	0.057
	Median	6	6	
	Min, max	1, 9	1, 10	
VAS (tinnitus annoyance) (0–10)	Mean ± std dev	5.3 ± 2.2	5.9 ± 2.4	0.108
	Median	5	6	
	Min, max	1, 10	0, 10	
VAS (tinnitus effect on life) (0–10)	Mean ± std dev	4 ± 2.6	4.3 ± 2.3	0.430
	Median	4	5	
	Min, max	0, 9	0, 9	
VAS (tinnitus effect on sleep) (0–10)	Mean ± std dev	3.2 ± 2.7	3.8 ± 2.8	0.754
	Median	3	3	
	Min, max	0, 9	0, 9	
VAS (tinnitus effect on concentration) (0–10)	Mean ± std dev	3.6 ± 2.7	3.7 ± 2.5	0.490
	Median	4	3	
	Min, max	0, 9	0, 8	
THI (0–100)	Mean ± std dev	37.1 ± 20.9	41.4 ± 21.4	0.100
	Median	34	42	
	Min, max	2, 76	0, 94	
HQ (0–42)	Mean ± std dev	13.3 ± 7.9	17.8 ± 8.4	<0.001*
	Median	14	18	
	Min, max	0, 36	0, 35	

*Wilcoxon test, asterisk represents a significant difference*.

**Figure 4:**
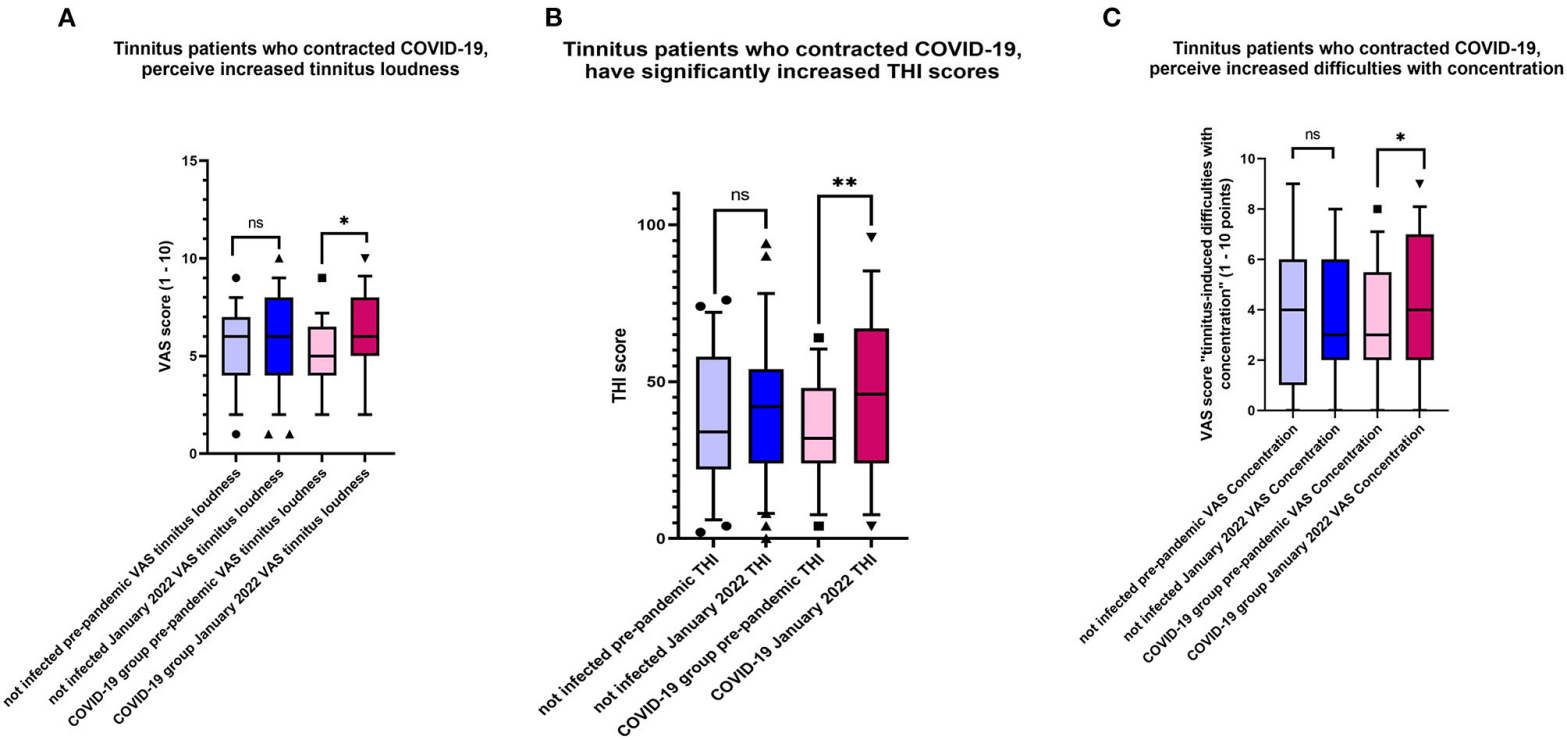
**(A)** Changes characteristic for tinnitus patients who contracted COVID-19. Plotted are the values indicating tinnitus loudness before the pandemic and January 2022. Shown is the box & whiskers plot (5–95 percentile), Wilcoxon paired test (*p = 0.0006; ns, not significant). **(B)** Changes characteristic for tinnitus patients who contracted COVID-19. Plotted are the values indicating THI scores before the pandemic and January 2022. Shown is the box & whiskers plot (5–95 percentile), Wilcoxon paired test (**p < 0.0001; ns, not significant). **(C)** Changes characteristic for tinnitus patients who contracted COVID-19. Plotted are the values indicating VAS scores “tinnitus-induced difficulties with concentration” before the pandemic and January 2022. Shown is the box & whiskers plot (5–95 percentile), Wilcoxon paired test (*p = 0.019; ns, not significant).

Within-group analysis of the tinnitus patients who contracted COVID-19 indicated that tinnitus loudness significantly increased after the disease (Table 4, Figure 4A). Moreover, the THI score increased significantly in that group following COVID-19 (Figure 4B), and these patients experienced more tinnitus-induced difficulties with concentration (Figure 4C).

**Table 4:** Scores of Tinnitus Handicap Inventory (THI), Hyperacusis Questionnaire (HQ), and the visual analog scales (VAS) in tinnitus patients who contracted COVID-19.

**Abbreviation (full name of test), and range (min-max)**		**Pre-COVID-19**	**Post-COVID-19**	***p***
VAS (tinnitus loudness) (0–10)	Mean ± std dev	5.2 ± 1.6	6.2 ± 1.9	0.006*
	Median	5	6	
	Min, max	2, 9	2, 10	
VAS (tinnitus annoyance) (0–10)	Mean ± std dev	5 ± 1.6	5.6 ± 2	0.086
	Median	5	5	
	Min, max	2, 9	2, 10	
VAS (tinnitus effect on life) (0–10)	Mean ± std dev	4.3 ± 2	4.4 ± 2.6	0.382
	Median	4	4	
	Min, max	0, 8	0, 10	
VAS (tinnitus effect on sleep) (0–10)	Mean ± std dev	3.4 ± 2.5	3.9 ± 2.9	0.066
	Median	3	4	
	Min, max	0, 8	0, 9	
VAS (tinnitus effect on concentration) (0–10)	Mean ± std dev	3.5 ± 2.2	4.2 ± 2.8	0.019*
	Median	3	4	
	Min, max	0, 8	0, 9	
THI (0–100)	Mean ± std dev	34.3 ± 15.4	45.9 ± 25.2	0.001*
	Median	32	46	
	Min, max	4, 64	4, 96	
HQ (0–42)	Mean ± std dev	13.8 ± 6.1	15.8 ± 8	0.169
	Median	12	16	
	Min, max	0, 27	2, 29	

*A paired difference test (Wilcoxon test) was used to calculate differences between before and after COVID-19 scores. Asterisk represents a significant difference*.

## Discussion

This study investigated the effect of COVID-19 on patients with chronic tinnitus diagnosed before the pandemic. The response to our first research question (“*does the infection with SARS-CoV-2 influence already existing tinnitus or hyperacusis?*”) was “*partially yes*,” as the infection influenced already existing tinnitus by increasing its loudness and THI scores but had not affected the hyperacusis scores. In response to our second research question (“*does the situation created by the pandemic influence already existing tinnitus or hyperacusis?*”), we demonstrated that hyperacusis significantly increases in uninfected tinnitus patients.

We found that in patients who have contracted COVID-19, tinnitus loudness and THI score increased significantly (Table 3). Our findings agree with Saunders et al., who also observed worsening tinnitus complaints in 23.1% of patients with preexisting tinnitus who contracted COVID-19 (12). However, in addition to the open question also used by Saunders et al., we used a validated instrument to measure the degree of tinnitus complaints and compared the values to the pre-pandemic ones. The reasons for worsening tinnitus complaints in infected tinnitus patients remain unclear. SARS-CoV-2 was recently shown to be capable of infecting the human inner ear and directly affecting the audio-vestibular system (38); however, no routine diagnostic tests can detect the audiovestibular infection. Still, the hypoxic and inflammatory mechanisms induced during the viral infection (39) could contribute to changes in the auditory system, and their involvement needs to be addressed in future research. COVID-19 has been associated with sudden sensorineural hearing loss (SSHL) (40); however, none of the patients included in our study reported SSHL. Unfortunately, because of the restricted access to our hospital during the pandemic, we were unable to examine our patients otologically, which leaves the possibility of an increase in tinnitus complaints associated with a hearing loss. In addition to the increased tinnitus loudness and THI scores, increased tinnitus-related difficulties with concentration were also noted among the infected tinnitus patients. Difficulties with concentration are a general sequel of COVID-19 (41, 42); therefore, our observation needs further follow-up to clarify if the difficulties are general or specifically related to increased tinnitus complaints. When we asked patients with COVID-19 whether their tinnitus complaints changed during the infection, many patients stated that tinnitus attracted less attention due to other problems they experienced. However, no improvement regarding tinnitus was mentioned. Although the scores indicating tinnitus annoyance, tinnitus effect on life, tinnitus effect on sleep, and HQ scores were higher after COVID-19, the differences before-after were not statistically significant. Additionally, as stated in the current literature, the relationship between COVID-19 and the onset of auditory symptoms or worsening of the preexisting ones might depend on the negative effects of the pandemic process and, therefore, should be established with care (12, 43).

In patients who have not contracted COVID-19, the THI scores and the scores for tinnitus loudness, tinnitus annoyance, tinnitus effect on life, tinnitus effect on sleep, and concentration increased after 2 years of the pandemic; however, that increase was not statistically significant (Table 3). Our data corroborate studies analyzing the effect of pandemic-related confinement on hearing loss and tinnitus in the Italian population (44) or the effect of the pandemic on tinnitus in the German population (45).

One of the most surprising findings of this study was that the hyperacusis scores measured with HQ significantly increased in the uninfected patients. During telephone interviews, most patients complained about hyperacusis, confirming the known association between hyperacusis and stress (46), with the pandemic situation acting as a stressor. Interestingly, a systematic review found that the general rate of anxiety increased during the pandemic compared to the pre-COVID-19 period (47). Anxiety can cause adverse effects in many systems. In their study of 3103 patients with chronic tinnitus, Beukes et al. reported that the severity of tinnitus might increase, reflecting the negative effect of COVID-19 on the emotional state (47). It is tempting to speculate that the pandemic could similarly increase hyperacusis complaints. In addition, there may be more than one reason for the worsening hyperacusis. As stated in the review of Tyler et al., many physiological and social factors may affect hyperacusis (48). In this case, the change in social life occurring during the pandemic may be one of the reasons for the increase in hyperacusis complaints. According to the environment-centered approach, the annoyance of everyday sounds is not always related to the loudness of the sound (49). The annoyance may also be associated with the social situation (50) or having perceived control over the noisy situation (51), which are the problems recently observed among the increased number of people working from home during the pandemic period. Most of the patients included in this study live in the city center. Considering that the number of people working from home is greater in city centers, they experienced different problems than those living in rural areas. Our patients, especially those with families with children, stated that they were exposed to more noise during the pandemic and tired while working from home. Uncontrollable noise during online meetings while working from home could be another reason increasing the complaint of hyperacusis. Considering that social norms also affect hyperacusis, it is likely more difficult to tolerate noises encountered at homes, such as created by neighbors', street, or electrical appliances. Some sounds that usually would not be bothersome can become annoying when the situation changes. Furthermore, financial worries, difficulties with sleep, and social isolation due to the COVID-19 pandemic might also affect mood (47). The non-increased hyperacusis complaints of patients who had contracted COVID-19 may be related to the symptoms of COVID-19 overriding the hyperacusis complaint.

Another hypothesis related to the increasing complaints of hyperacusis during the pandemic is the central gain mechanism (52, 53). The enhancement of neural gain due to reduced auditory input may have increased the complaint of hyperacusis in people living in cities who are lonely, participate in less noisy leisure activities, or are exposed to less traffic noise during the pandemic period. The mechanism of hyperacusis is still the subject of intense research and remains unclear. Therefore, more research is needed in this area.

Our study is not free of limitations. Although we collected a detailed medical history and used valid and reliable questionnaires, clinical evaluation of the patients in January 2022 was not performed due to the COVID-19 pandemic, and the patient's hearing threshold or psychoacoustic tinnitus features were not assessed. One of the crucial missing tests is the pure tone audiometry, which we hope to perform as soon as possible to assess possible changes in the hearing abilities. Another limitation is the vaccination status of the patients. In this study, the type and number of vaccinations were very heterogeneous, leaving their impact on tinnitus unknown. A more extensive study should address this issue in the future.

## Conclusion

In the current study, significantly increased tinnitus complaints were observed in patients who underwent COVID-19. In contrast, patients who had not contracted COVID-19 had increased hyperacusis complaints. The findings show that COVID-19 infection and the pandemic-created situation can influence the already existing tinnitus and hyperacusis.

## Data Availability Statement

The raw data supporting the conclusions of this article will be made available by the authors, without undue reservation.

## Ethics Statement

The studies involving human participants were reviewed and approved by The Clinical Research Ethics Committee of Istanbul Medeniyet University (approval number 2021/0665). The patients/participants provided their written informed consent to participate in this study.

## Author Contributions

ME: study design, data collection, and drafting the manuscript. AM: study design, drafting the manuscript, and data analysis. SC: study design and drafting manuscript. MK: study design, study conceptualization, and manuscript revisions. AS: study conceptualization, data analysis, visualization, and manuscript drafting and revisions. All authors contributed to the article and approved the submitted version.

## Conflict of Interest

The authors declare that the research was conducted in the absence of any commercial or financial relationships that could be construed as a potential conflict of interest. The reviewer HH is currently organizing a Research Topic with the author AS.

## Publisher's Note

All claims expressed in this article are solely those of the authors and do not necessarily represent those of their affiliated organizations, or those of the publisher, the editors and the reviewers. Any product that may be evaluated in this article, or claim that may be made by its manufacturer, is not guaranteed or endorsed by the publisher.
